# Case of Enciphaloid Cancer of Kidney

**Published:** 1878-04

**Authors:** W. T. Belfield


### CASE OF ENCEPHALOID CANCER OF KIDNEY.

By Dr. Belfield.

Mrs. B., aged 63, was admitted to the hospital Jan. 23, 1878. Her health had been uniformly excellent until two years previously, when she began to suffer occasional pain, brief but sharp, in the right iliac and lumbar regions. This pain was especially severe when the bladder was full and during the act of micturition. With the lapse of time these pains occurred more frequently; a little blood was occasionally noticed in the urine; and finally a well-defined tumor became apparent just to the right of the umbilicus. Meanwhile her general health declined; she lost flesh rapidly ; her symptoms became gradually aggravated, the pain, size of tumor and quantity of blood in the urine constantly increasing.

On admission, the patient was very feeble and emaciated. The urine contained about 10 per cent. of albumen ; large granular tube-casts, and numerous cells widely differing in size and shape—*i. e.*, atypical. A considerable admixture of blood, sometimes clotted, was constantly present. A semi-fluctuating, somewhat movable, globular tumor, painful but not tender, and as large as an infant's head, occupied the right iliac and umbilical regions.

The patient's general health steadily declined ; facial erysipelas supervened, and death occurred Feb. 2.

Upon opening the abdominal cavity, the viscera were found displaced, distorted, but matted together by firm adhesions into one chaotic mass. Closer inspection and dissection resolved this mass into a large central tumor, to which were adherent the pyloric end of the stomach, the head of the pancreas, the right lobe of the liver and the gall-bladder; the ascending and transverse colon, and several folds of small intestines. There appeared to be nothing resembling a kidney on the right side. There was no appearance of general peritonitis.

The tumor itself was of oval shape, about nine inches long and six inches in its conjugate diameter. It extended from the lower surface of the liver to the right iliac fossa, lying in contact with the posterior abdominal walls and with the spinal column, its anterior surface being covered by the peritoneum. Beneath the latter membrane was a close interlacement of veins, many of which were as large as a goose quill. The ureter was traced into the central portion of the mass. Many retro-peritoneal and mesenteric glands were much enlarged and quite soft.

Disseminated through the tumor, but especially numerous and large near the surface, were cysts which varied in size from a pea to a walnut, and from which there escaped upon puncture a thin, milky liquid, containing much *débris* of tissue.

Section revealed marked differences in appearance between different portions of the mass. The outer or cortical portion (that part farthest from the ureter) was of light brown color, and had suffered considerable loss of tissue in the formation of cysts. At the depth of one and a half inches, the color changed abruptly to a dirty white. Both layers of tissue but especially the outer were quite pultaceous, crumbling readily under the finger. There was no gross appearance of renal structure except at the lower extremity of the mass. Here, separated by sharp lines of demarcation from the softened tissues was a mass as large as a walnut of what appeared to be normal kidney.

*Microscopy.*—A section taken from the cortical portion of the kidney consisted of a mass of cells without any visible intercellular substance, divided into irregular groups by a fibrous framework, or rather permeated here and there by delicate threads of fibrous tissue. The cells varied in size from a blood corpuscle to a bladder epithelial cell, and exhibited the greatest diversity of contour, being circular, oval, polygonal, candate, etc. The nuclei and nucleoli were generally very distinct; many of the cells contained each two nuclei. In most of them the protoplasm was quite granular, sometimes obscuring the nuclei; in some the protoplasm was more or less completely disintegrated, leaving the nuclei nearly or quite free. Almost every field contained a considerable number of oil-drops.

The fibrous tissue of the growth was very scanty and presented no especial features. Sections from the deeper portion of the tumor, and from the enlarged lymphatic glands exhibited essentially the same features, except that the cellular elements were in an advanced state of fatty degeneration.

A section from the small part of apparently healthy kidney showed considerable degeneration. The highly granular condition of all the anatomical elements rendered somewhat difficult the differentiation between malpighian tufts, vessels and tubules. In consequence of this cloudiness the nuclei of the tubular epithelium were quite indistinct; moreover, numerous oil-drops attested the advanced stage of the degenerative process.

